# Innovative treatment modalities for urinary incontinence: a European survey identifying experience and attitude of healthcare providers

**DOI:** 10.1007/s00192-017-3339-y

**Published:** 2017-04-21

**Authors:** Arnoud W. Kastelein, Maarten F. A. Dicker, Brent C. Opmeer, Sonia S. Angles, Kaisa E. Raatikainen, Joan F. Alonso, Diana Tăut, Olavi Airaksinen, Linda D. Cardozo, Jan-Paul W. R. Roovers, on behalf of the WOMEN-UP consortium

**Affiliations:** 1 Department of Obstetrics and Gynaecology, Academic Medical Centre, Room F4-240, Meibergdreef 9, 1105 AZ Amsterdam, The Netherlands; 2 Clinical Research Unit, Academic Medical Centre, Amsterdam, The Netherlands; 3 Department of Obstetrics and Gynaecology, Hospital Clinic, Barcelona, Spain; 4 Department of Obstetrics and Gynaecology, Kuopio University Hospital, Kuopio, Finland; 5 Department of Automatic Control, Biomedical Engineering Research Centre, Universitat Politècnica de Catalunya, Barcelona, Spain; 6 Department of Psychology, Babeş-Bolyai University, Cluj-Napoca, Romania; 7 Department of Rehabilitation Medicine, Kuopio University Hospital, Kuopio, Finland; 8 Department of Obstetrics and Gynaecology, King’s College Hospital, London, UK

**Keywords:** Urinary incontinence, Serious gaming, Biofeedback, Pelvic floor muscle training

## Abstract

**Introduction and hypothesis:**

Urinary incontinence is a common condition in women, with a reported prevalence ranging from 25% to 51%. Of these women, an estimated 38% suffer from stress urinary incontinence (SUI). A European research consortium is investigating an innovative system based on information and communication technology for the conservative treatment of women with SUI. When introducing a new intervention, implementation barriers arise and need to be identified. Therefore, we investigated healthcare providers’ experience with and attitude towards innovative care options.

**Methods:**

We performed an online survey to assess (1) the characteristics and practice of healthcare providers, (2) current protocols for SUI, (3) current use of biofeedback, and (4) knowledge about serious gaming. The survey was sent to members of professional societies in Europe (EUGA), UK (BSUG) and The Netherlands (DPFS).

**Results:**

Of 341 questionnaires analyzed (response rate between 18% and 30%), 64% of the respondents had access to a protocol for the treatment of SUI, and 31% used biofeedback when treating patients with SUI. However, 92% considered that biofeedback has a clear or probable added value, and 97% of those who did not use biofeedback would change their practice if research evidence supported its use. Finally, 89% of respondents indicated that they had no experience of serious gaming, but 92% considered that it could be useful.

**Conclusions:**

Although inexperienced, European urogynecologists and physical therapists welcome innovative treatment options for the conservative treatment of SUI such as portable wireless biofeedback and serious gaming. Scientific evidence is considered a prerequisite to incorporate such innovations into clinical practice.

## Introduction

Urinary incontinence is a common condition in women, with a reported prevalence ranging from 25% to 51% [1]. Of these women, an estimated 38% suffer from stress urinary incontinence (SUI) [2], a condition that has a major impact on the quality of life [3]. Pelvic floor muscle training (PFMT) is an effective treatment for women with mild or moderate SUI [4] and is recommended as part of first-line conservative management programs for women with SUI [5]. However, because the efficacy of PFMT is directly related to adherence, poor compliance can significantly reduce the cure rates with this treatment [6, 7]. Healthcare providers try to support adherence with more frequent patient visits, which has been proven to be effective but makes the intervention more costly [8–11].

A European research group, the WOMEN-UP consortium, collaborates on an innovative intervention to expose women with SUI to the benefits of pelvic floor physiotherapy, optimize adherence and reduce costs. This novel intervention involves a wireless vaginal biofeedback device and an abdominal belt, both with surface electromyography sensors, connected via Bluetooth to a smartphone with access to ‘serious games’. Both vaginal and abdominal biofeedback are obtained, enabling patients to improve their training technique. Exercise performance and results can be monitored by patients and their therapists through an online web portal with two-way messaging functionality.

In serious gaming, interactive training games are used for a primary purposes other than pure entertainment, that is to improve knowledge, skill or attitude with the added value of fun and competition [12, 13]. Serious games have been successfully used in, for instance, rehabilitation programs [14] and for promoting health behavior [15]. In the WOMEN-UP project, serious games will be employed to make PFMT more appealing and thereby possibly improve adherence. In these serious games, contracting and relaxing the pelvic floor operates a game on a smartphone via a Bluetooth biofeedback signal. In addition, to evaluate this innovative approach in terms of clinical and cost effectiveness, professionals’ attitudes towards pelvic physiotherapy, biofeedback and serious gaming need to be known. Implementation of new treatment modalities can be hampered by multiple factors, including healthcare providers’ attitudes towards and knowledge of the innovative technology. It is important to identify, assess and tackle these factors in a timely manner to optimize the intervention and facilitate its application in practice.

For example, sufficient knowledge of PFMT, biofeedback and serious gaming, and their therapeutic value seem essential. The lack of scientific evidence and the absence of a standardized protocol describing the treatment leave healthcare providers hesitant to use new treatment modalities. Therefore, as a first step, the current protocol and data on experience with and attitudes towards biofeedback combined with serious gaming need to be collected and described for the different European countries. It is also important to understand the attitudes of those who do not use biofeedback, and the conditions under which they would be willing to use such a treatment modality. Suggestions and criticisms should be taken into account and acted upon to minimize the risk of problems during subsequent implementation.

Therefore, the aim of this research was to investigate whether and when this innovative solution for the conservative treatment of urinary incontinence would fit in professionals’ daily practice by performing a Europe-wide survey.

## Materials and methods

We designed a survey that comprised 57 multiple choice and open questions. The questions were designed mainly to assess (1) the background characteristics of healthcare providers, (2) their current use of biofeedback devices, (3) their attitudes and expectations regarding biofeedback devices, and (4) their attitudes and expectations regarding serious gaming for the treatment of SUI. Given the nature of our research method, ethical approval was not required. All members of the European Urogynaecology Association (EUGA), the British Society of Urogynaecology (BSUG) and the Dutch Pelvic Floor Society (DPFS) were sent a link to an online electronic questionnaire built using SurveyMonkey (SurveyMonkey Inc., Palo Alto, CA; www.surveymonkey.com). The members of these societies consist mainly of urogynecologists. Recipients received the link by e-mail once, followed by reminders. Subsequently, the data entered were saved automatically on a server and extracted after the survey had been closed.

### Characteristics of respondents and their practice

These were assessed by questions concerning age, gender, training, experience and workplace.

### Current protocol and daily clinical practice

The questionnaire included 25 questions that addressed daily clinical practice. Healthcare providers were asked whether a protocol was available in their clinic for the treatment of urinary incontinence. Questions also addressed the primary and preferred treatment for mild and moderate SUI, other available therapeutic options, and if these included physical therapy. Healthcare providers were also asked whether and how patients were referred in their region and whether patients were reimbursed for physiotherapy treatment. Differences among European countries regarding referral and reimbursement were assessed.

### Current use of biofeedback

The questionnaire included 16 questions that addressed experience with and attitude towards biofeedback in more detail. To assess the current use of biofeedback among healthcare providers, they were asked “Do you currently use biofeedback devices in your practice?” They were then asked which device they used and the reason for their choice. They were also asked if they believed that biofeedback could be of additional benefit in the treatment of SUI. To evaluate possible improvement in currently available devices, the main disadvantages encountered by therapists were evaluated. Specific ideas for improvement could be mentioned. Healthcare providers who did not use biofeedback were asked the reason why, and what they would improve or change in currently available devices. They were asked what they considered the success rate of biofeedback treatment should be before they would be willing to use it in daily clinical practice. Again, reimbursement and the indications necessary for possible reimbursement were also evaluated.

### Knowledge about serious gaming

The questionnaire included five questions addressing experience with and attitude towards serious gaming. These included “Do you have any experience in using serious gaming?”. If respondents did have experience, a question followed about what serious gaming is used for and if they thought serious games could be useful for the self-management of SUI. Respondents could choose ‘age’, ‘education’, ‘computer skills’, ‘motivational levels’ and ‘contact with other patients using the same intervention’ as factors they considered most likely to positively contribute to the use of serious gaming.

### Statistical analysis

Descriptive statistics are used to present the demographic variables. Categorical data are presented as frequencies and percentages. Non-normally distributed continuous data are presented as medians and interquartile ranges. Normally distributed continuous data are presented as means and standard deviations. The normality of continuous data was tested using the Kolmogorov-Smirnov test for normality. All data were analyzed using SPSS, version 23.0 for Windows (IBM Corp., Armonk, NY).

## Results

Of a total of 1,844 questionnaires submitted, 341 were returned (response rate between 18% and 30%). As shown in Table 1, 306 (90%) of the respondents were urogynecologists and 17 (5%) were pelvic floor muscle therapists from 26 European countries. Many worked at university hospitals (118, 35%) or teaching hospitals (132, 39%). Most respondents were based in The Netherlands (117), UK (60), Turkey (25), Czech Republic (14) and Poland (13). Not all respondents answered all questions. Therefore, the results are presented as the numbers and percentages of respondents who answered each question.

**Table 1:** Demographic and baseline characteristics

Variable	Value
Total number of respondents, *n* (%)	341 (100)
Age (years), median (IQR)	49 (42–58)
Sex, *n* (%)	
Male	177 (51.9)
Female	142 (41.6)
Background, *n* (%)	
Urogynecologists	306 (89.7)
Pelvic floor muscle therapists	17 (5.0)
Countries, *n*	26
Workplace, *n* (%)	
University hospital	118 (34.6)
(Other) teaching hospital	132 (38.7)
Nonteaching hospital	42 (12.3)
General practice/healthcare center	19 (5.6)
Pelvic floor physiotherapy unit	6 (1.8)
Working experience (years), median (IQR)	15 (8–23)

### Current protocol and daily clinical practice

**Fig. 1:**
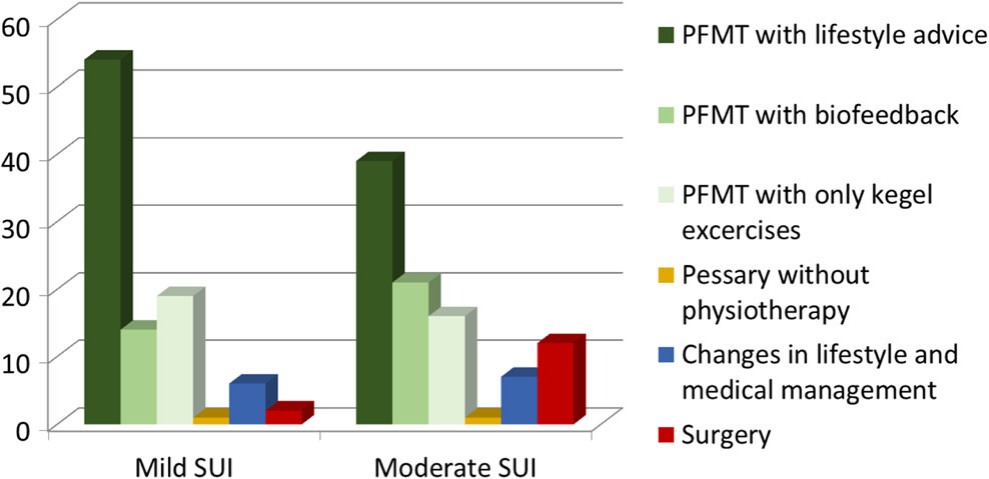
Protocols for mild and moderate SUI throughout Europe

A total of 204 respondents answered the question regarding the current treatment of urinary incontinence. The majority (130, 64%) indicated that a protocol was available. In most protocols, the primary treatment consisted of lifestyle advice and PFMT in patients with both mild (54% of protocols used) and moderate (39% of protocols used) SUI (Fig. 1). A minority of respondents treated these patients differently with, for instance, a pessary or surgery.

The most common route of referral of patients with incontinence to gynecologists was either by general practitioner (117 of 185 who answered this question, 63%) or by self-referral (34, 18%). There was a clear difference between European countries. The 73 Dutch and 40 British respondents who answered this question indicated there was no self-referral to a gynecologist. However, the 11 Turkish and 14 Greek respondents who answered this question indicated that self-referral was the most common route of referral to a gynecologist. A proportion of patients do not see a gynecologist before referral to a physiotherapist. Of the 185 respondents who answered this question, more than half (106, 57%) indicated that patients were not always seen by a gynecologist prior to referral to a physiotherapist. These respondents were based in Belgium, Germany, Ireland, The Netherlands, Poland, Spain, the UK and Switzerland. Just over half of respondents (95/173, 55%) who answered questions about reimbursement indicated that patients were reimbursed for physiotherapy treatment. There were clear differences between countries: 70% of the 73 Dutch respondents who answered this question indicated that between five and ten sessions were reimbursed, but all Polish respondents that answered this question (n=6/13) indicate that there was no reimbursement. The estimated percentage of patients undergoing surgery after PFMT was 41–60% according to the majority of respondents (67, 39%) of the 173 who answered this question.

### Biofeedback

**Fig. 2:**
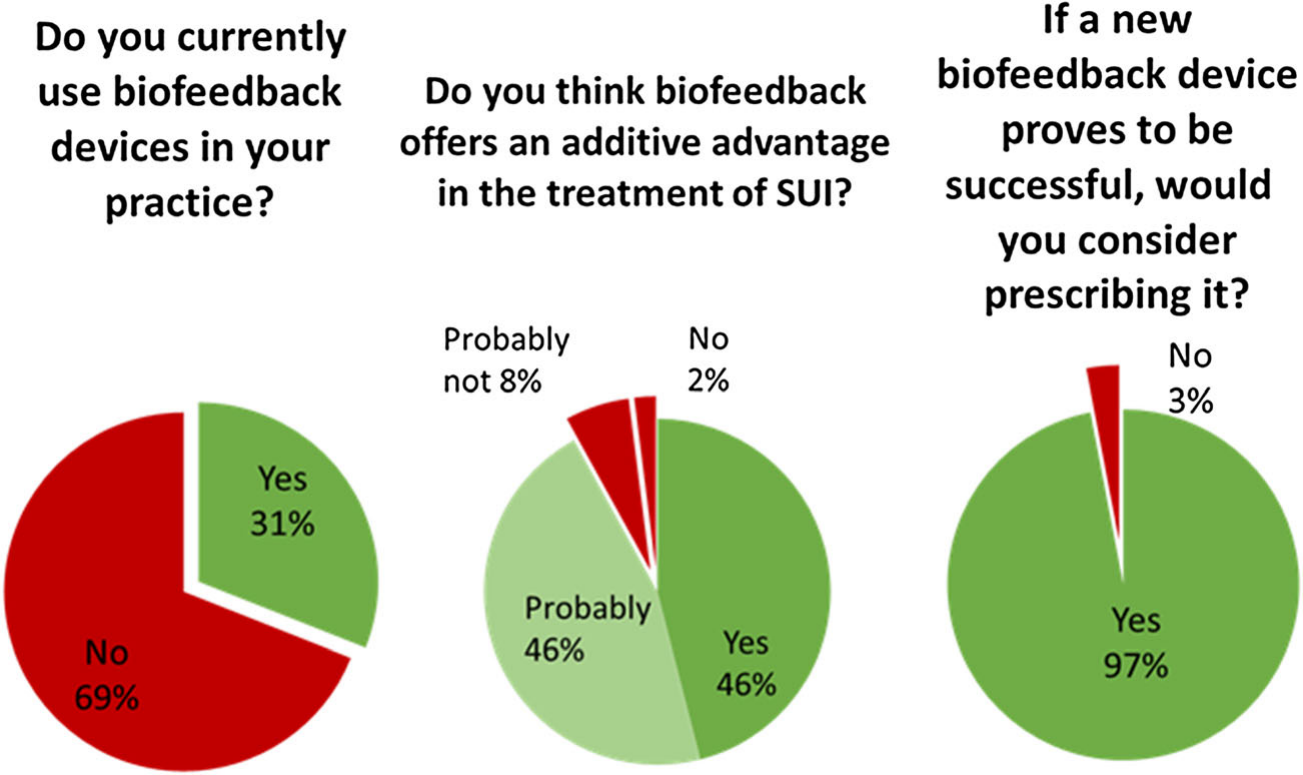
Current use of biofeedback

As shown in Fig. 2, of 298 respondents who answered the question regarding biofeedback, a minority (92, 31%) currently used this in their practice. There was a difference in the use of biofeedback between gynecologists (29%) and pelvic floor therapists (79%). Of 309 respondents who answered the question regarding the additive advantage of biofeedback in the treatment of SUI, 143 (46%) considered that there is a clear advantage and 143 (46%) considered that there is a probable advantage. Of 200 respondents who answered the question as to whether they would change their practice and offer a biofeedback device if research evidence supported its use, almost all (193, 97%) indicated that would change their practice.

Of 80 respondents who answered the question regarding reimbursement for biofeedback devices, a minority (28, 35%) indicated that patients were fully or partially reimbursed. This differs from reimbursement for physiotherapy treatment, for which, as stated above, 55% of respondents (95/173) indicated that patients were reimbursed.

Table 2 shows the suggestions from respondents regarding improvement in currently available biofeedback devices. Of 241 respondents who answered the question regarding the main advantages of biofeedback, 68 (28%) were of the opinion that biofeedback can increase patient motivation. Also, 55 respondents (23%) and 31 respondents (13%) considered that biofeedback can provide objective feedback on the quality and frequency, respectively, of training, and 38 (16%) considered that biofeedback can increase adherence to treatment. The major disadvantages of biofeedback were considered by the respondents to be the cost to the patient and the fact that scientific evidence supporting its use is lacking. Of 190 respondents who answered the question regarding the minimum success rate of PFMT combined with biofeedback, most (88, 46%) indicated that this has to be between 41% and 60%. There were no significant differences in age between respondents who did and those who did not use biofeedback.

**Table 2:** Suggestions on changes to currently available biofeedback systems in answer to the question: “What would you like to improve on the existing device you are currently working with or those you are familiar with?”

Suggestion	No. (%) of respondents answering this question
More accessible: available to all, mobile application, for home use and self-management, portable, connection to smartphone	14 (29)
Cost: lower cost	8 (17)
Different sizes, more comfort, more user friendly	6 (13)
Recording abdominal muscle activity	1 (2)
More contact between patient and professional	1 (2)
Nothing needs to be improved	6 (13)
Cannot say, do not know	3 (6)
Not valid	9 (16)

### Serious gaming

**Fig. 3:**
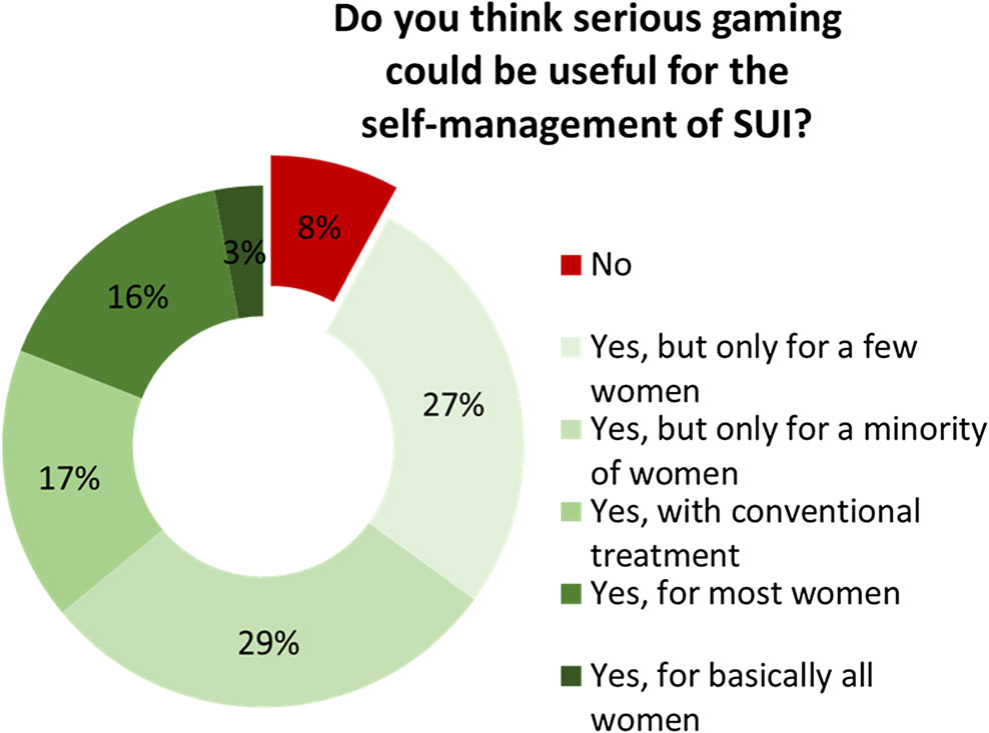
Usefulness of serious gaming for the self-management of SUI

Only a few respondents (30/268, 9%) reported experience with serious gaming. However, as shown in Fig. 3, of the 268 respondents who answered the question regarding the usefulness of serious gaming for the self-management treatment of SUI, 244 (92%) considered that it could be useful. Yet many emphasized the limited therapeutic value in certain patient groups and indicated that serious gaming would not be beneficial in all patients. ‘High levels of motivation’ and ‘age’ were considered pivotal for successful use of serious gaming.

## Discussion

We conducted an online survey to assess current practice of gynecological healthcare providers and to investigate their experience with and attitude towards pelvic physiotherapy, biofeedback and serious gaming. This European-wide survey showed that urogynecologists and pelvic floor physiotherapists have limited experience with these eHealth applications. Only a third of respondents used biofeedback in the treatment of SUI and very few (11%) had experience with serious gaming. Nonetheless, almost all respondents would welcome innovative solutions to improve existing conservative treatment modalities for SUI. In their opinion they could be of added value for their current therapeutic practice, but they needed clinical evidence to support this.

This is the first inventory of current clinical practice and attitudes regarding innovative technologies for the treatment of SUI in Europe. One of the strengths of this study is the design of the survey that was conducted by researchers from different European countries including gynecologists from university clinics in The Netherlands, Spain and Finland. Also, responses were received from professionals from 26 different countries. This geographical spread contributed to a diverse picture that provides some insight into different practices across Europe.

The questionnaire could be criticized for its relative simplicity. To adequately document local conditions, referral patterns and standard pathways of care for patients with different severities of SUI, a far more detailed and open-ended questionnaire would have been required. However, in order to minimize the nonresponse rate, the length of the questionnaire was minimized and clear, straightforward items were conceived, that may not have adequately reflected the variety in local practice. Despite our efforts to maximize the response rate, including several reminders, still only one-fifth of submitted questionnaires were returned. We do not know the exact response rate because some professionals are members of more than one urogynecology association and may therefore have received the questionnaire more than once. For example, some members of the Dutch and British pelvic floor societies will have received the survey twice, since they also received it as EUGA members. As a consequence, the response rate may vary between 18% and 30% depending on the degree of overlap in society membership. Another limitation is the seemingly limited response from some countries and the fact that the majority of respondents were based un The Netherlands and UK. We do not know the response rates for individual countries, since the characteristics of the members of professional societies were not available. Because respondents were mainly based in The Netherlands and the UK, it is questionable if the survey can be generalized to urogynecologists and pelvic floor physiotherapists across Europe. Therefore, the results of this research should be interpreted with caution.

It is remarkable that a third of European urogynecologists and pelvic floor physiotherapists did not have protocols available in their clinic for the treatment of SUI. The exact reason of this is unknown, but this is a rather worrying finding and suggests room for improvement in standard clinical practice. The absence of a protocol will likely result in unwanted variation between treatments, depriving some patients of optimal evidence-based care. When a novel treatment modality has been proven effective, it might lay the foundation for a Europe-wide protocol for the treatment of SUI. However, differences between nations need to be kept in mind.

We have addressed differences between countries in terms of referral patterns and reimbursement. Especially in those countries where there is no or little reimbursement for physiotherapy and where the infrastructure is relatively poor and/or the geographical spread large, the use of eHealth might be of added value. When access to healthcare is restricted, the need for alternatives becomes greater. Especially when commercially available and thus priced affordably, such an innovative treatment modality for SUI might have significant potential. In other fields of medicine, eHealth has been proven of value in motivation and education in women’s healthcare and self-management of disease [12–17]. Some literature also suggests that adding biofeedback to PFMT improves outcome by increasing compliance rates, enhancing utilization and improving training technique [18–22]. However, evidence is insufficient to provide strong recommendations about the best way to approach PFMT [11, 22, 23]. In particular, data on long-term outcomes are lacking [24–26]. Respondents seemed aware of this, because only a third of urogynecologists currently used biofeedback. However, the fact that more than 90% considered that it could have added advantage suggests that respondents did believe in the potential benefits. When asked what needs to be improved, many stressed the importance of more scientific evidence. Feedback-mediated exercise has been proven of value in rehabilitation after stroke. Researchers describe prolonged endurance in training and greater improvement in certain aspects of motor function, as well as very high patient motivation and acceptance [14, 27]. These findings are promising for the utilization of biofeedback in urogynecology.

Besides insufficient evidence, respondents mentioned other important reasons for not using biofeedback, such as the cost and reimbursement. This drives the need for a more easily accessible, self-administered and commercially available system that is affordable for practices and patients. As shown in Table 2, when asked to suggest how currently available devices could be improved, most respondents suggested a more user-friendly, accessible, affordable self-management system to use at home. Apparently, there is an unmet clinical need for a better and cost-effective self-management system.

Evaluation of the use of serious gaming showed similarities to the use of biofeedback. Serious games have only recently found their way into clinical practice, although evidence has shown efficacy in divergent patient populations in other fields of medicine [12–16]. A review by the WOMEN-UP consortium recently accepted for publication also suggests that serious gaming is efficacious [28]. Nevertheless, only a small percentage of respondents had some experience with serious gaming. As almost all respondents indicated they would use serious gaming when available, lack of familiarity might be responsible. The solution to this problem is appropriate education and training of healthcare professionals [29]. Many respondents also underscored the limited therapeutic value in certain patient groups, and indicated that the use of serious gaming should be restricted to certain patients (Fig. 3). As serious gaming may require some computer or technical skill, this is probably partially true, and also because research has shown that a substantial proportion of the population never use health apps and many stop using them [30]. However, the literature suggests that the attitude of healthcare providers is a significant factor in the acceptance and efficient use of information technology in practice. Successful implementation actually depends on the therapist, rather than on the patient [29].

### Conclusions

With current rapid developments in information technology, healthcare professionals must keep up to date to make the most of these auspicious opportunities. Therefore, the overall positive attitude of European urogynecologists and pelvic floor physiotherapists towards this issue is promising. Their lack of familiarity with innovative care options does not seem to be based on reluctance or ignorance and, in fact, they are apparently willing to change practice and welcome new treatment modalities.

Respondents ideally wanted a more user-friendly, accessible, affordable self-management system to use at home. The WOMEN-UP consortium aims to develop a system that can meet these demands. However, evidence proving the efficacy of such a treatment modality is crucial. This makes the clinical trial that the WOMEN-UP consortium are undertaking of great importance. This nonblinded, randomized controlled trial will include 300 patients in The Netherlands, Spain and Finland and will compare standard care PFMT with the innovative treatment modalities.
